# Synthesis and Properties of Inositol Nanocapsules

**DOI:** 10.3390/ma14195481

**Published:** 2021-09-22

**Authors:** Songping Mo, Yuanhong Li, Shaofei Shan, Lisi Jia, Ying Chen

**Affiliations:** Guangdong Provincial Key Laboratory on Functional Soft Condensed Matter, School of Materials and Energy, Guangdong University of Technology, Guangzhou 510006, China; mosp@ustc.edu (S.M.); 2112002067@mail2.gdut.edu.cn (Y.L.); 2111902184@mail2.gdut.edu.cn (S.S.); jialisi@gdut.edu.cn (L.J.)

**Keywords:** phase-change material, nanoencapsulation, inositol, thermal energy storage

## Abstract

Sugar alcohols are phase-change materials with various advantages but may suffer from leakage during applications. In this study, inositol nanocapsules were synthesized at various conditions, including the amount of precursors and the time for adding the precursors. The effects of synthesis conditions on the properties of the nanocapsules were studied. The morphology, chemical composition, microstructure, phase-change characteristics and size distribution of the nanocapsules were investigated by scanning electron microscope (SEM), Fourier transform infrared spectroscopy (FT-IR), transmission electron microscope (TEM), differential scanning calorimeter (DSC) and a zeta potential analyzer. The results confirm that inositol was well-encapsulated by an SiO_2_ shell. The shell thickness increased, while the supercooling degree of the nanocapsules decreased with increasing time for adding the precursors. In order to obtain nanocapsules with good morphology and phase-change characteristics, the time for adding the precursors should increase with the amount of precursors. The nanocapsules with the best properties exhibited high melting enthalpy, encapsulation ratio and energy storage efficiency of 216.0 kJ/kg, 83.1% and 82.1%, respectively. The size of the nanocapsules was remarkably affected by the triethoxysilane (TES) amount.

## 1. Introduction

Thermal energy storage is an effective method that coordinates the contradiction between energy supply and demand in time and space [1]. Latent heat storage stores heat in a phase-change material (PCM), providing much higher energy storage density, smaller volume requirement and smaller temperature swing compared to sensible heat storage [2].

Sugar alcohols are good phase-change material candidates due to high phase-change enthalpy, high volumetric energy density, non-flammability as well as a proper phase-change temperature range [3]. In spite of these desirable properties of PCMs, leakage of melted PCMs, volume and pressure variations during phase transformation, corrosiveness and low thermal conductivities are their major drawbacks, which limit applications of PCMs [4]. In order to overcome these problems, techniques of utilizing micro/nano-encapsulated phase-change materials (MPCMs/NPCMs) in thermal energy storage systems have been developed [5]. 

Many attempts have been carried out to encapsulate PCMs with organic polymer shell materials, such as polymethyl methacrylate [6], polycarbonate [7], melamine formaldehyde [8]. However, the application of MPCMs with polymeric shells is severely restricted in some cases due to their toxicity, flammability [9], and low thermal conductivity [10,11]. Though the thermal conductivity can be enhanced by nanoparticles [12], the poor thermal stability of organic shell materials at high temperature remained a problem [11]. Therefore, in recent years, much attention has been focused on inorganic shell materials, such as calcium carbonate (CaCO_3_) [13], organo-silica [14], and silicon dioxide (SiO_2_) [15], for their high thermochemical stability, environmental friendliness and low toxicity.

As a kind of water-soluble PCM, sugar alcohol MPCMs/NPCMs are much more difficult to be prepared compared with oil-soluble PCMs [16], since most of fabricating methods are based on oil-in-water emulsions in which water is the continuous phase. Thus, there are only a few studies on sugar alcohol MPCMs/NPCMs. Salaün et al. [17] reported the synthesis of xylitol microcapsules in the urethane-urethane shell via interfacial polycondensation and found that core/shell weight ratio influences the shell formation mechanism, the mean diameter and morphology of the microcapsules. Makuta et al. [18] synthesized xylitol microcapsules with average size of 3–5 μm and a latent heat of 115 kJ/kg by using a cyanoacrylate monomer. In the past years, researchers explored inorganic shell materials to encapsulate sugar alcohols. Wang et al. [19] obtained polysiloxane capsules containing erythritol by an ultraviolet-assisted hydrolysis method, and found that the supercooling degree remarkably decreased compared with pure erythritol. Pethurajan et al. [20] reported that D-mannitol was encapsulated into a SiO_2_ shell material using the sol-gel technique with tetraethyl orthosilicate (TEOS) as a silica precursor. The encapsulation ratio and efficiency were 89.60% and 85.02%, respectively, when the pH was maintained at between 2.6 and 3.0. He et al. [16] reported the synthesis of D-mannitol nanocapsules via a facile sol-gel method in which SiO_2_ was used as the shell material. The resulting nanoencapsulated D-mannitol demonstrated good thermal reliability and phase-change performance.

Although the limited studies demonstrate the possibility of encapsulation of sugar alcohols, to the best of our knowledge, no study has been reported on the synthesis of inositol MPCMs/NPCMs. Inositol has a high latent heat of 224.5 kJ/kg and a melting temperature of 261.8 °C [21], which makes a promising PCM for various medium temperature applications such as solar thermal energy storage. Moreover, synthesis conditions have great impacts on the phase-change performance of MPCMs/NPCMs. The reason is that the shell material can be a nucleating agent for the PCM core, thus affects the phase-change characteristics of the MPCMs/NPCMs. Cao et al. [22] found that microcapsules prepared with larger ratio of formaldehyde to melamine (F:M ratio) exhibited a large supercooling degree which was associated with the liquid-triclinic crystalline transition induced by homogeneous nucleation, while the supercooling can be largely eliminated when the F:M ratio reduced to 1.25. Yang et al. [23] reported that microcapsules with the thickest shell materials exhibited the minimum supercooling phenomena since the shell materials acted as nucleating agents. However, the effects of synthesis conditions on the properties of sugar alcohol MPCMs/NPCMs have not been reported. Therefore, in this study, inositol was nanoencapsulated at various synthesis conditions, including amount of the precursors and the time for adding the precursors. The effects of synthesis conditions on the properties of the nanocapsules, including the morphology, chemical composition, microstructure, phase-change characteristics and size distribution, were investigated.

## 2. Materials and Methods

### 2.1. Materials

Inositol (analytical reagent grade of 98% purity) and triethoxysilane (TES, analytical reagent grade of 98% purity) were obtained from Aladdin Chemistry Co., Ltd., Shanghai, China. Tetraethoxysilane and cyclohexane were purchased from Tianjin Damao Chemical Reagent Factory, China. Span 80 and Tween 80 of chemical purity were purchased from Sigma-Aldrich (Shanghai) Trading Co., Ltd, Shanghai, China. All the chemicals were used as received from the suppliers without any further purification.

### 2.2. Preparation of Inositol Nanocapsules

The inositol nanocapsules were synthesized by the sol-gel method. First, the water phase was prepared by adding 5.1 g inositol and 0.1 g Tween 80 into 15 mL deionized water and then stirred using a magnetic stirrer at 800 rpm while the temperature was controlled at 50 °C. The oil phase was prepared by adding 0.6 g of Span 80 and 65 mL cyclohexane, and stirred under continuous agitation at the room temperature for 10 min. Then, the water phase was added into the oil phase and continuously stirred at 800 rpm for half an hour at 50 °C. As a result, a stable water-in-oil (W/O) emulsion was formed. 

**Figure 1:**
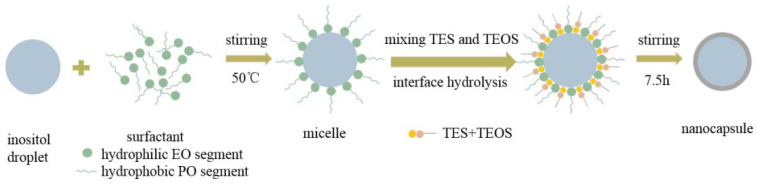
Schematic formation of the inositol@SiO_2_ nanocapsules via the sol-gel process.

The encapsulation reaction was conducted in a three-necked flask. The TES and TEOS were mixed together in a beaker to form the precursor solution. Ten types of nanocapsules were prepared, as listed in Table 1.The precursor solution was injected into the W/O emulsion drop-by-drop through a syringe pump (TYD01-02-CE, Baoding Refu Fluid Technology Co., Ltd., Hebei, China), while the mixture was continuously stirred at 800 rpm for 7.5 h at 50 °C. Figure 1 schematically illustrates the procedure for encapsulation of inositol within the silica nanocapsules by the hydrolysis and condensation of TEOS between the interfaces of the emulsion via a sol-gel route. The aqueous solution was mixed with the oil phase containing the emulsifier, which resulted in a stable W/O emulsion. The TEOS hydrolyzed to form the sol solution. Then, the condensation reaction between the orthosilicic acid took place to form the oligomers. The oligomers polymerized to build the SiO_2_ shell on the surface of the inositol nano-droplets. Finally, the resultant nanocapsules were washed by cyclohexane three times and freeze-dried at −40 °C for 12 h.

### 2.3. Characterization of the Nanocapsules

The chemical structure of the pure inositol, SiO_2_ and the nanocapsules was analyzed using a Fourier transform infrared spectrometer (FT-IR, Nicolet 6700, Thermo Scientific, Waltham, MA, USA) over the wavelength range from 400 to 4000 cm^−1^. The morphologies of the pure inositol and the nanocapsules were observed by a scanning electron microscope (SEM, SU8010, Hitachi Inc., Tokyo, Japan). The core and shell structure of the nanocapsules was investigated using a transmission electron microscope (TEM, JEM-2100, JEOL, Tokyo, Japan). The phase-change properties and thermal energy storage performance of the pure inositol and the nanocapsules, such as melting and freezing temperature and latent heats, were obtained using a differential scanning calorimetry (DSC3 STAR, Mettler Toledo, Zurich, Switzerland) under a nitrogen atmosphere from 100 to 250 °C at a heating/cooling rate of 10 °C/min. The particle size distribution of the nanocapsules was measured using a particle size and zeta potential analyzer (Zetasizer Nano ZS, Beckman Coulter, Inc., Miami, USA).

## 3. Results and Discussion

### 3.1. Morphology of the Nanocapsules

**Figure 2:**
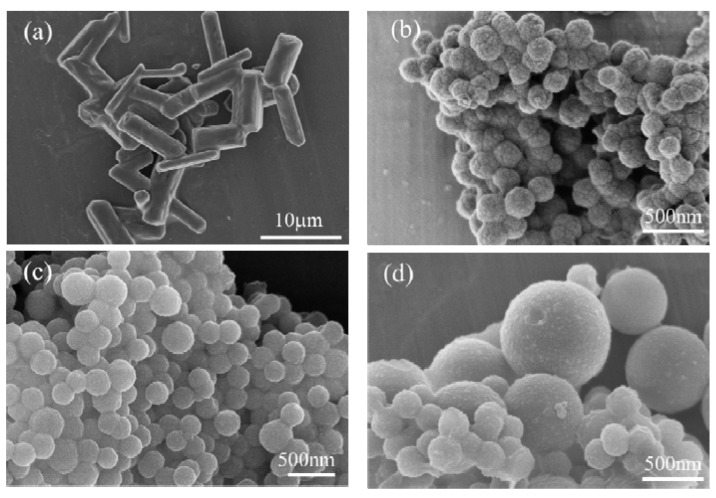

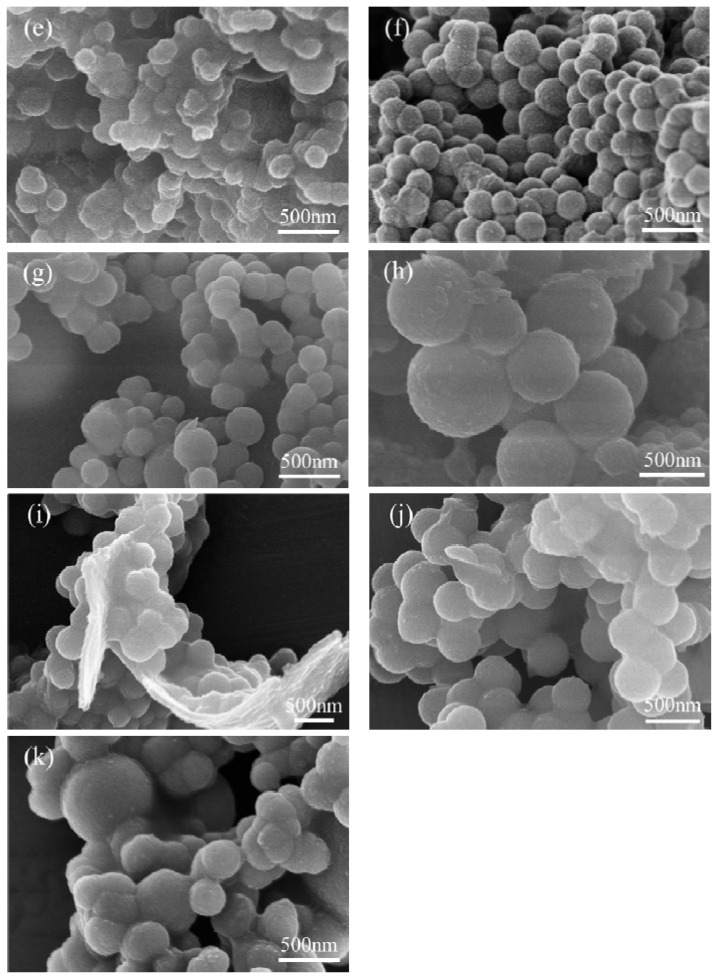
SEM micrographs of inositol and the nanocapsules: (**a**) IN; (**b**) NeIN1; (**c**) NeIN2; (**d**) NeIN3; (**e**) NeIN4; (**f**) NeIN5; (**g**) NeIN6; (**h**) NeIN7; (**i**) NeIN8; (**j**) NeIN9; (**k**) NeIN10.

The morphologies of the inositol and the nanocapsules were shown in Figure 2. As shown in Figure 2a, the inositol particles were irregular in shape and size with an average size of several micrometers. Figure 2b–k shows that the nanocapsules were typically spheres with an average size of approximately several hundred nanometers, which were much smaller than that of the inositol particles. The reason is that the inositol was uniformly dispersed into nanodroplets during the emulsification. The nanocapsules with a small diameter may improve heat transfer efficiency because of the larger surface-to-volume ratio compared to the inositol particles. Besides, the morphologies of the nanocapsules vary from the different experimental synthesis conditions. Among the nanocapsules, the nanocapsules of NeIN2, NeIN5, NeIN6, and NeIN9 were well-dispersed, spherically-shaped and with uniform size. The influences of various synthesis conditions on the morphology, properties and implications on applications of the nanocapsules are discussed in detail in the following sections.

### 3.2. Chemical Characterization of the Nanocapsules

**Figure 3:**
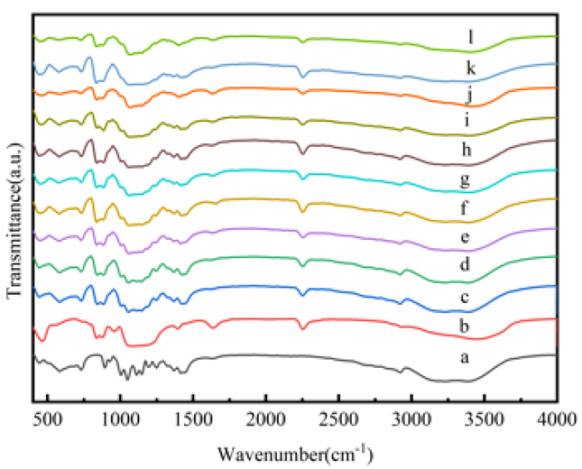
FT-IR spectra of (**a**) inositol, (**b**) SiO_2_ and the nanocapsules (**c**) NeIN1; (**d**) NeIN2; (**e**) NeIN3; (**f**) NeIN4; (**g**) NeIN5; (**h**) NeIN6; (**i**) NeIN7; (**j**) NeIN8; (**k**) NeIN9; (l) NeIN10.

FT-IR spectroscopy was used to investigate the interaction between the inositol and SiO_2_ of the nanocapsules. The FT-IR spectra of the inositol, SiO_2_ and the nanocapsules are presented in Figure 3. In the SiO_2_ spectrum, the absorption peaks at 476 cm^−1^, 806 cm^−1^ and 1112 cm^−1^ represent the bending vibration, symmetric and asymmetric stretching vibrations of the Si-O-Si group, while the absorption peak at 962 cm^−1^ is attributed to the bending vibration of the Si-OH group [10]. In the inositol spectrum, the absorption peaks at 732 cm^−1^ and 1427 cm^−1^ are attributed to the out-of-plane and in-plane bending vibration peaks of the -OH group, respectively. The peaks at 1000 cm^−1^, 1051 cm^−1^ and 1110 cm^−1^ are assigned to the stretching vibration of the C-OH group. The peak at 2925 cm^−1^ corresponds to the stretching vibration of the -CH group. The absorption peak at 1638 cm^−1^ and the broad peak at 3450 cm^−1^ are associated with the bending vibration and asymmetric stretching vibration of the -OH group of the absorbed water [23]. In the nanocapsule spectrum, all the main characteristic peaks of inositol and SiO_2_ can be found, and no other new peaks were observed, indicating that inositol was physically encapsulated by SiO_2_, and there was no chemical reaction between the inositol core and the SiO_2_ shell of the nanocapsules.

### 3.3. Phase-Change Properties of the Nanocapsules

**Figure 4:**
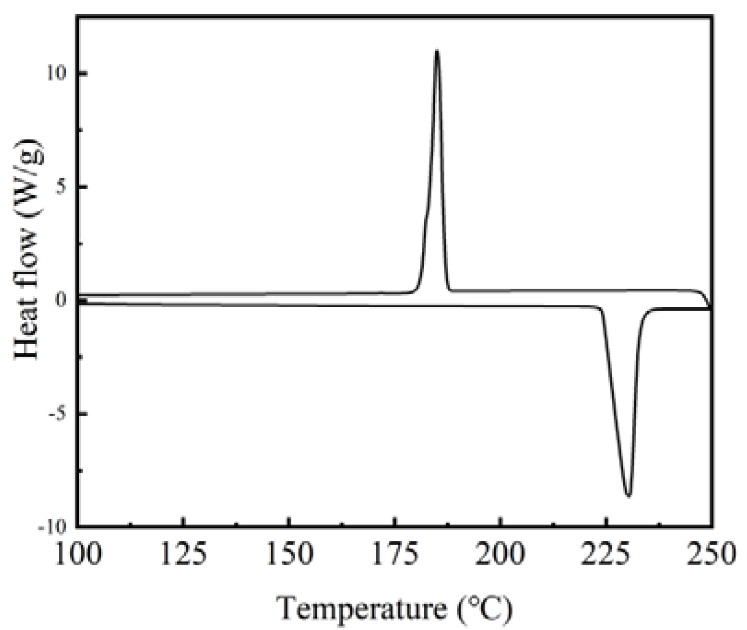
DSC curves of the inositol.

Figure 4 illustrates the melting and solidifying processes of the inositol. Both the DSC melting and solidifying curves present a single endothermic or exothermic peak. The DSC results of the inositol are listed in Table 2. The difference between the melting temperature, solidifying temperature, latent heat of melting and solidifying of the inositol obtained in the present study and those reported in the literature is 0.4 °C, 10 °C, 0.9 kJ/kg and 0.6 kJ/kg, respectively. As a result, the supercooling degree, which is defined as Δ*T* = *T*_m_ − *T*_s_, exhibited a difference of 9.6 °C between the present result and that in the literature [21]. The values of the phase-change temperature and latent heat are comparable to those in the literature, except for the solidifying temperature. The reason may be due to the randomness of supercoiling [24].

The encapsulation ratio (*R*) and energy storage efficiency (*E*) are defined to describe the phase-change properties of the nanocapsules, which can be calculated using the following equations:

*R* = ∆*H*_m,NeIN_/∆*H*_m,IN_ (1)

*E =* (∆*H*_m,NeIN_
*+* ∆*H*_S,NeIN_)*/*(∆*H*_m,IN_
*+* ∆*H*_S,IN_), (2)

where ∆*H*_m,NeIN_ and ∆*H*_m,IN_ are the melting enthalpies of the nanocapsules and pure inositol, respectively; ∆*H*_s,NeIN_ and ∆*H*_s,IN_ are the solidifying enthalpies of the nanocapsules and pure inositol, respectively. The DSC results of the nanocapsules are listed in Table 3. As shown, the melting and solidifying temperatures of the nanocapsules were in the range of 226–229 °C and 175–184 °C, respectively. Compared with the pure inositol, the melting temperature of the nanocapsules increased by about 2–3 °C while the solidifying temperature decreased by 7–15 °C. The increase in the melting temperature might be due to the hydrogen-bond interaction between the inositol and the SiO_2_ shell. The decrease in the solidifying temperature is attributed to the increased seed deactivation with decreased sample size [25]. The enthalpies of the nanocapsules are lower than those of the inositol since SiO_2_ is an inert material with no phase-change in the temperature range and only the inositol core in the nanocapsules absorbs/releases thermal energy during the heating/cooling processes. Among the nanocapsule samples, NeIN6 exhibited the highest melting enthalpy, solidifying enthalpy, encapsulation ratio and energy storage efficiency of 216.0 kJ/kg, 160.8 kJ/kg, 83.1 % and 82.1 %, respectively. The influences of various synthesis conditions on the performance of the nanocapsules are analyzed in the following sections.

### 3.4. Effect of Time for Adding the Precursors

**Figure 5:**
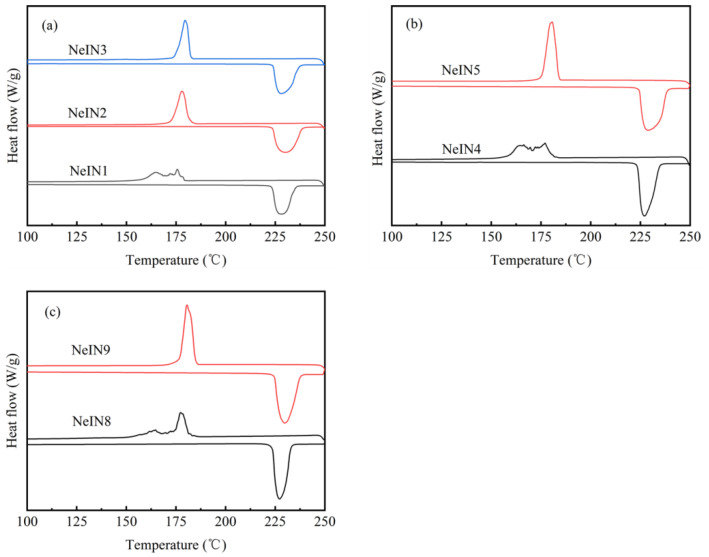
DSC curves of the nanocapsules with various conditions: (**a**) TES/TEOS = 5 mL/9 mL; (**b**) TES/TEOS = 5 mL/10 mL; (**c**) TES/TEOS = 7 mL/9 mL.

Figure 5 shows the DSC curves of the nanocapsules that were prepared at the same amount of TES and TEOS but different time during which the precursors were added, *t_a_*. As shown in Figure 5a, when TES/TEOS = 5/9, the solidifying curve of NeIN1 with *t*_a_ of 20 min shows a multiple-peaks shape, but those of NeIN2 and NeIN3 with *t*_a_ of 30 min and 40 min, respectively, exhibit a single-peak shape. As shown in Figure 5b,c, when TES/TEOS = 5/10 and 7/9, the solidifying curves of NeIN4 and NeIN8 with *t*_a_ of 30 min show multiple peaks; however, as *t*_a_ was increased to 40 min, NeIN5 and NeIN9 show a single-peak shape. These results indicate that enough reaction time is required so that the solidifying curves transform from multiple-peaks to a single-peak. The different DSC peak spectrum may be attributed to the effect of nucleation induction by the shell of the nanocapsules. It was proposed that nucleation on the droplet walls or homogeneous nucleation dominates in the nucleation of small droplets in PCM emulsions [25]. Similarly, for the nucleation of the nanocapsules, nucleation on the shells, or homogeneous nucleation, dominates. Therefore, when *t*_a_ was insufficient for the formation of the shells (NeIN1, NeIN4 and NeIN8), both nucleation on the shells and homogeneous nucleation dominates, resulting in multiple peaks in the solidifying curves and higher supercooling degree (Table 3). By contrast, when *t*_a_ was sufficient for the formation of the shells (NeIN2, NeIN3, NeIN5 and NeIN9), nucleation on the shells dominates, resulting in single peak in the solidifying curves.

The SEM micrographs of NeIN1, NeIN2 and NeIN3 with the same TES/TEOS amount of =5 mL/9 mL but increasing *t*_a_ are shown in Figure 2b–d. All the nanocapsules are not broken. Some of the NeIN1 nanocapsules adhered together. The reason is that when the *t*_a_ is too short, some of the precursors do not fully react to form SiO_2_. As a result, on the one hand, some of the inositol was not encapsulated so that the encapsulation ratio and energy storage efficiency of NeIN1 were relatively low (Table 3) and the nanocapsules were attached to each other by the unencapsulated inositol (Figure 2b); on the other hand, the multi-component shell results in a multi-peak DSC solidifying curve (Figure 5a). When the *t*_a_ is long enough, which is the case for NeIN2, the nanocapsules are well-dispersed, spherically-shaped, with a uniform size of 150–250 nm and relatively high encapsulation ratio and energy storage efficiency (Table 3). NeIN3 are mixtures of small nanocapsules with a diameter of about 200 nm surrounded by big nanocapsules of 600 nm. Furthermore, there are some debris on the surface of NeIN3, suggesting that some of the inositol was not encapsulated so the encapsulation ratio and energy storage efficiency were lower than those of NeIN2 (Table 3). The reason may be that the formation rate of the shell was too low when *t*_a_ was too long for the relatively small amount of precursors.

The SEM micrographs of NeIN4, NeIN5, NeIN8 and NeIN9, are shown in Figure 2e, Figure 2f, Figure 2i and Figure 2k, respectively. When the amount of the precursors TES/TEOS increased to 5 mL /10 mL and 7 mL /11 mL, more reaction time was required. Therefore, when the time for adding the precursors was less than sufficient (30 min for NeIN4, NeIN8), the precursors do not fully react to form SiO_2_. As a result, similar to the case of NeIN1, on the one hand, NeIN4 and NeIN8 had a relatively low encapsulation ratio and energy storage efficiency (Table 3) and the nanocapsules were attached to each other by the unencapsulated inositol; on the other hand, the multiple peaks in the DSC solidifying curves were observed (Figure 5) for the same reason as that for NeIN1. On the contrary, if *t*_a_ is sufficient (e.g., 40 min for NeIN5 and NeIN9), the precursors can completely react to form the SiO_2_ shell, so NeIN5 and NeIN9 have an improved dispersed spherically-shaped appearance with more uniform size and the solidifying curves change from multiple-peaks to a single-peak (Figure 5). 

**Figure 6:**
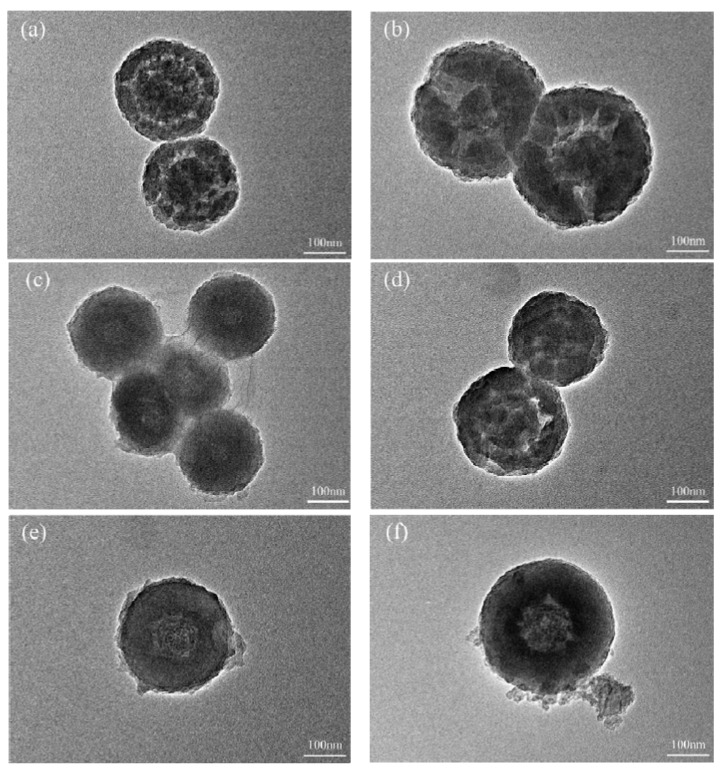

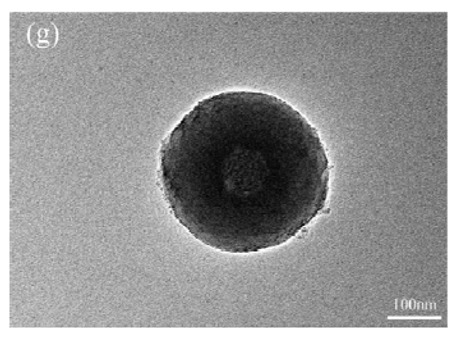
TEM micrographs of the nanocapsules: (**a**) NeIN1; (**b**) NeIN2; (**c**) NeIN3; (**d**) NeIN4; (**e**) NeIN5; (**f**) NeIN8; (**g**) NeIN9.

Moreover, as shown in Figure 6, the core-shell structure was observed for the nanocapsules. For the same TES/TEOS amount, the thickness of the shells of the nanocapsules increases with increasing *t*_a_. There is some debris around NeIN8 (Figure 6f), possibly being the unencapsulated inositol due to insufficient *t*_a_. However, little debris can be found around NeIN9, which further demonstrates that as the *t*_a_ increases, the shell was better formed to protect the core material and prevent its leakage. Furthermore, according to the data from Table 2, the supercooling degree of the nanocapsules decreases with increasing *t*_a_, suggesting that the SiO_2_ shell could act as a nucleating agent, which induces the nucleation of inositol.

### 3.5. Effect of TEOS Amount

Three sets of control experiments were conducted to study the effects of TEOS amount on the morphology and phase-change characteristics of the synthesized nanocapsules: (1) The amount of TEOS was 9 mL, 10 mL, 11 mL, respectively for synthesizing NeIN3, NeIN5 and NeIN6, when *t*_a_ was 40 min and the amount of TES was 5 mL; (2) The amount of TEOS was 9 mL, 10 mL, respectively for NeIN9 and NeIN10, when *t*_a_ was 40 min and the amount of TES was 7 mL; (3) The amount of TEOS was 9 mL, 10 mL, respectively for NeIN2 and NeIN4, when *t*_a_ was 30 min and the amount of TES was 5 mL.

**Figure 7:**
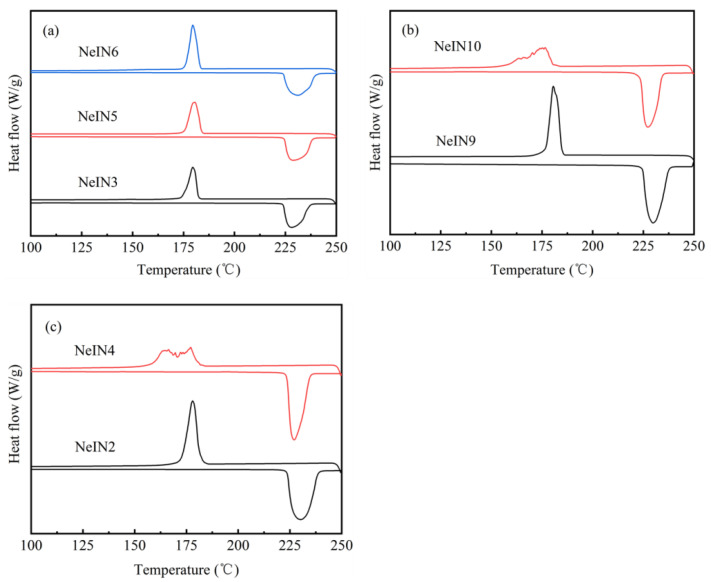
DSC curves of the nanocapsules with various conditions: (**a**) NeIN3, NeIN5 and NeIN6; (**b**) NeIN9 and NeIN10; (**c**) NeIN2 and NeIN4.

The DSC curves of the nanocapsules are presented in Figure 7. All the melting curves of the nanocapsules showed a single-peak shape, while the solidifying curves of NeIN10 and NeIN4 show multiple peaks, and the solidifying curves of the other nanocapsules show a single-peak shape. As shown in Figure 7b,c, when the time for adding the precursors and the amount of TES are constant, excess TEOS leads to multiple peaks in the solidifying curves of the nanocapsules. The results suggest that adding too much TEOS within relatively short *t*_a_ leads to inadequate formation of the shells. As a result, both nucleation on the shells and homogeneous nucleation occur, resulting in multiple peaks in the DSC solidifying curves, higher supercooling degree, lower encapsulation ratio and energy storage efficiency (Table 3).

The SEM micrographs of NeIN3, NeIN5 and NeIN6 are presented in Figure 2d, f and g, respectively. The reason for NeIN3 having uneven size and a certain degree of agglomeration is that the inositol cannot be fully encapsulated since the amount of TEOS was too small. For NeIN5, when the TEOS amount was high enough, the inositol can be well-encapsulated so the nanocapsules were well-dispersed with a uniform size and spherical morphology. Further increasing the TEOS amount (NeIN6) may result in excess silica formation which also causes aggregation of the nanocapsules.

The SEM micrographs of NeIN9, NeIN10, NeIN2 and NeIN4 are shown in Figure 2c and Figure 2e, Figure 2j, Figure 2k, respectively. With the proper TEOS amount, NeIN9 and NeIN2 have spherical shapes and uniform size distribution. However, when there is excess TEOS, which is the case for NeIN10 and NeIN4, there was extra silica which connected between the nanocapsules, and the particle size of NeIN10 was uneven.

The results indicate that excess or insufficient amount of the TEOS used in the synthesis process will lead to deterioration of the size uniformity and morphology of the nanocapsules. Only when the amount of TEOS is moderate, spherical nanocapsules with uniform particle size and good dispersion can be obtained. In addition, throughout comprehensive analysis with the DSC curves, compared with the multiple-peaks solidifying curve of NeIN4 and NeIN10, NeIN2 and NeIN9 exhibited a single-peak solidifying curve, suggesting that the multiple DSC solidifying peaks can be attributed to the excess formation of the shell of the nanocapsules.

### 3.6. Effect of TES Amount

Three sets of control experiments were also set up to study the influence of TES amount on the morphology and phase-change characteristics of the nanocapsules: (1) The amount of TES was 5 mL, 6 mL, 7 mL, respectively for NeIN3, NeIN7 and NeIN9, when *t*_a_ was 40 min and the TEOS amount was 9 mL; (2) The TES amount was 5 mL and 7 mL, respectively for NeIN5 and NeIN10, when *t*_a_ was 40 min and the TEOS amount was 10 mL; (3) The TES amount was 5 mL and 7 mL, respectively for NeIN2 and NeIN8, when *t*_a_ was 30min and the TEOS amount was 9 mL.

**Figure 8:**
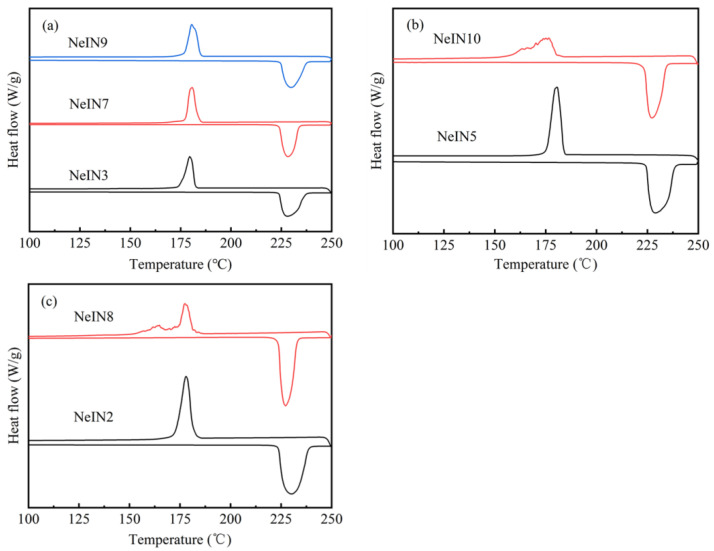
DSC curves of the nanocapsules with various conditions: (**a**) NeIN3, NeIN7 and NeIN9; (**b**) NeIN5 and NeIN10; (**c**) NeIN2 and NeIN8.

The DSC curves of the nanocapsules are presented in Figure 8. All the melting curves of the nanocapsules show a single-peak shape, while the solidifying curves of NeIN10 and NeIN8 showing multiple peaks, and the solidifying curves of the other nanocapsules showing a single-peak shape. Similar to the case of excess TEOS amount, adding too much TES within a relatively short *t*_a_ leads to the inadequate formation of the shell, which resulted in the coexistence of shell-induced nucleation and homogeneous nucleation, corresponding to the multiple DSC solidifying peaks.

The SEM micrographs of NeIN3, NeIN7 and NeIN9 are presented in Figure 2d, Figure 2h and Figure 2j, respectively. With insufficient TES, NeIN3 showed uneven size. With enough TES, NeIN7 and NeIN9 were uniform in size and the uniformity of the nanocapsules increased with an increasing amount of TES.

The SEM micrographs of NeIN5, NeIN10, NeIN2 and NeIN8 are shown in Figure 2c, Figure 2f, Figure 2i, and Figure 2k, respectively. With proper TES amount, NeIN5 and NeIN2 have regular spherical structures with excellent particle size uniformity and dispersion. With excess TES, there are extra silica connecting the nanocapsules of the NeIN10 and NeIN8. The particle size of NeIN10 was uneven, while NeIN8 aggregated into agglomerates with poor dispersion uniformity.

Compared with the results of the samples with different TEOS amount, it can be seen that the TES amount affects the nanocapsules shape and particle size in the similar way. Moreover, for all the synthesized samples, spherical nanocapsules with better dispersion and higher uniformity had a single-peak DSC solidifying curve (e.g., NeIN5 and NeIN2).

Furthermore, it was found that the three parameters, i.e., the time for adding the precursors, the TEOS amount and TES amount have combined effects on the properties of the nanocapsules, and thus the selection of the three parameters affects one another. In order to obtain nanocapsules with good properties, the time for adding the precursors should increase with the amount of TEOS and TES, while, for a given time for adding the precursors, the amount of TEOS and TES should not be too much or too little.

### 3.7. Particle Size Distribution of the Nanocapsules

**Figure 9:**
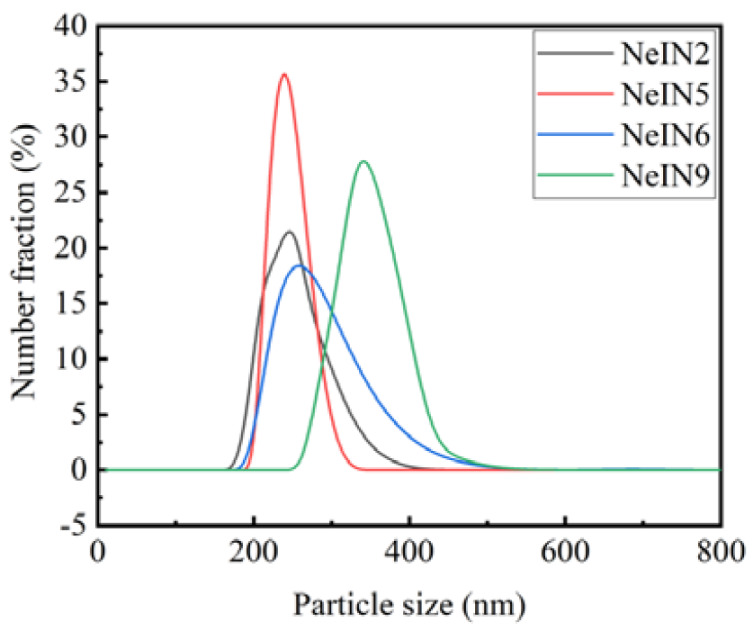
Particle size distribution of the nanocapsules.

The size distribution of the nanocapsules with outstanding performance obtained in this study (i.e., NeIN2, NeIN5, NeIN6 and NeIN9) was measured and the results are presented in Figure 9. The size of the nanocapsules was in the range of 190–400, 200–300, 200–400, 250–450 nm with the peak maximums of 250, 242, 265 and 334 nm for NeIN2, NeIN5, NeIN6 and NeIN9, respectively. The results are consistent with the particle sizes of the nanocapsules shown in the SEM analysis (Figure 2). The results indicate that NeIN9 nanocapsules are bigger than the other nanocapsules (NeIN2, NeIN5 and NeIN6). By comparing the synthesis parameters of these nanocapsules (Table 1), it was found that the TES amount remarkably affected the size of the nanocapsules, while the effects of the TEOS amount and the time for adding the precursors were neglectable.

To sum up, the experimental results show that nanocapsules that were well -dispersed spherically-shaped with uniform size (i.e., NeIN2, NeIN5, NeIN6 and NeIN9) also exhibited single-peak solidifying characteristics, low supercooling degree, high encapsulation ratio and high energy storage efficiency. These properties favor the application of the nanocapsules as phase-change latent heat storage materials. Therefore, the morphology of the nanocapsules is an important indicator of the functional properties and application potentials of the materials.

## 4. Conclusions

In this paper, inositol SiO_2_ nanocapsules were synthesizes by a sol-gel method. The effects of synthesis conditions on the morphology, phase-change characteristics and particle size distribution of the nanocapsules were investigated. The main conclusions are as follows.

(1) Inositol was successfully encapsulated by SiO_2_ shell and no chemical reaction between the inositol core and SiO_2_ shell was observed.
(2) The shell thickness of the nanocapsules increased while the supercooling degrees decreased with increasing time for adding the precursors. The results suggest that the SiO_2_ shell could act as a nucleating agent which promotes the nucleation of the inositol core.
(3) The nanocapsules that were well-dispersed, spherically-shaped with uniform size also exhibited good phase-change properties. In order to obtain good morphology and phase-change properties, the time for adding the precursors should increase with the amount of the precursors. Excess or insufficient time for adding the precursors, TEOS amount or TES amount resulted in reduced encapsulation performance, including poor size uniformity, multiple nucleation, decreased encapsulation ratio and energy storage efficiency. NeIN6 exhibited the best performance with melting enthalpy, encapsulation ratio and energy storage efficiency of 216.0 kJ/kg, 83.1 % and 82.1 %, respectively.
(4) The TES amount remarkably affected the size of the nanocapsules, while the effects of the TEOS amount and the time for adding the precursors were neglectable.

## Figures and Tables

**Table 1:** Synthesis parameters for the preparation of the nanocapsules.

Sample	TES (mL)	TEOS (mL)	Time for Adding Precursors (min)
NeIN1	5	9	20
NeIN2	5	9	30
NeIN3	5	9	40
NeIN4	5	10	30
NeIN5	5	10	40
NeIN6	5	11	40
NeIN7	6	9	40
NeIN8	7	9	30
NeIN9	7	9	40
NeIN10	7	10	40

**Table 2:** Phase-change properties of the inositol.

Sample	*T*_m_ (°C)	*H*_m_ (kJ/kg)	*T*_s_ (°C)	*H*_s_ (kJ/kg)	Δ*T* (°C)
Present	224.9	260.9	191.4	198.0	33.5
Ref. [21]	224.5 ± 0.2	261.8 ± 0.1	181.4 ± 0.5	198.6 ± 0.2	43.1 ± 0.7

**Table 3:** Phase-change properties of the nanocapsules.

Sample	*T*_m_ (°C)	*H*_m_ (kJ/kg)	*T*_s_ (°C)	*H*_s_ (kJ/kg)	*R* (%)	*E* (%)	*ΔT* (°C)
NeIN1	227.1	167.2	175.9	96.0	64.1	57.4	51.2
NeIN2	228.6	192.6	179.6	131.4	73.8	70.6	48.9
NeIN3	226.5	188.6	181.2	126.6	72.3	68.7	45.3
NeIN4	226.2	176.2	177.3	100.6	67.5	60.3	48.9
NeIN5	227.4	210.4	182.4	144.7	77.2	75.4	45.0
NeIN6	228.9	216.0	183.1	160.8	83.1	82.1	45.8
NeIN7	227.1	191.0	182.2	126.5	73.3	69.2	44.9
NeIN8	226.4	121.4	177.9	67.3	46.5	41.1	48.5
NeIN9	227.2	196.6	182.3	143.3	75.3	74.0	44.9
NeIN10	226.3	165.1	176.8	85.5	63.3	54.6	49.5

## Data Availability

Data sharing is not applicable for this article.
